# Comparison of Properties of Medial Entorhinal Cortex Layer II Neurons in Two Anatomical Dimensions with and without Cholinergic Activation

**DOI:** 10.1371/journal.pone.0073904

**Published:** 2013-09-12

**Authors:** Motoharu Yoshida, Arthur Jochems, Michael E. Hasselmo

**Affiliations:** 1 Faculty of Psychology, Mercator Research Group - Structure of Memory, Ruhr-University Bochum, Bochum, Germany; 2 International Graduate School of Neuroscience, Ruhr-University Bochum, Bochum, Germany; 3 Center for Memory and Brain, Department of Psychology and Graduate Program for Neuroscience, Boston University, Boston, Massachusetts, United States of America; University of Exeter, United Kingdom

## Abstract

Mechanisms underlying grid cell firing in the medial entorhinal cortex (MEC) still remain unknown. Computational modeling studies have suggested that cellular properties such as spike frequency adaptation and persistent firing might underlie the grid cell firing. Recent in vivo studies also suggest that cholinergic activation influences grid cell firing. Here we investigated the anatomical distribution of firing frequency adaptation, the medium spike after hyperpolarization potential (mAHP), subthreshold membrane potential oscillations, sag potential, input resistance and persistent firing, in MEC layer II principal cells using in vitro whole-cell patch clamp recordings in rats. Anatomical distributions of these properties were compared along both the dorso-ventral and medio-lateral axes, both with and without the cholinergic receptor agonist carbachol. We found that spike frequency adaptation is significantly stronger in ventral than in dorsal neurons both with and without carbachol. Spike frequency adaptation was significantly correlated with the duration of the mAHP, which also showed a gradient along the dorso-ventral axis. In carbachol, we found that about 50% of MEC layer II neurons show persistent firing which lasted more than 30 seconds. Persistent firing of MEC layer II neurons might contribute to grid cell firing by providing the excitatory drive. Dorso-ventral differences in spike frequency adaptation we report here are opposite from previous predictions by a computational model. We discuss an alternative mechanism as to how dorso-ventral differences in spike frequency adaptation could contribute to different scales of grid spacing.

## Introduction

Grid cells found in the medial entorhinal cortex (MEC) layer II are suggested to play an important role in spatial navigation [1]. Grid cell firing has been proposed to be generated within the MEC local circuit [2]–[5]. Cellular properties of MEC neurons such as subthreshold membrane potential oscillations (SMPOs) [2], [3], [6], resonance [6]–[8], input resistance [9], firing frequency adaptation [10] and persistent firing [11] may play an important role in grid cell firing. The SMPO frequency, the resonance frequency and the input resistance have been reported to vary systematically along the dorso-ventral (DV) axis and are suggested to underlie the gradient of spacing of grid cell firing fields at different positions along the DV axis [6], [9], [12], [13]. A recent study on HCN1 channel KO mice with reduced subthreshold membrane potential oscillations (SMPOs) and resonance has shown a wider spacing of grid cells, further suggesting that cellular properties play crucial roles in grid cell firing [14]. However, what determines the gradient of grid cell spacing remains unknown since the grid spacing difference was maintained in this study [14]. Moreover, the anatomical gradient of many of the cellular properties of MEC layer II cells have not been studied along the medio-lateral (ML) axis [7].

Recent in vivo intracellular recordings have shown that grid cells have properties predicted by both continuous attractor models and oscillatory interference models [15], suggesting that a hybrid of both models may explain experimental observations better [16]. Of note is the sustained depolarization seen as the animal crossed the grid field [15] which was predicted by continuous attractor models. Such depolarization in continuous attractor models, are usually supported by recurrent excitatory connections. However, excitatory recurrent connections are virtually nonexistent in the MEC layer II [17] [18], [19] and it remains unknown where such excitatory drive comes from. Recent work has shown that inactivation of the medial septum, which provides cholinergic projections to the MEC, disrupts grid cell activity [20], [21]. Lesions of the basal forebrain cholinergic system disrupt idiothetic navigation in mice [22]. Although cholinergic modulation of SMPO along the DV axis has been reported [23], the differential effect of cholinergic activation on other cellular properties along the DV axis remains unknown. Cholinergic modulation may also provide depolarization drive through persistent firing [16]. However, persistent firing in MEC layer II neurons has not been studied thoroughly.

In this paper, we investigated cellular properties of MEC layer II neurons at different two dimensional (DV and ML) anatomical positions across the extent of the MEC. Properties were tested with and without the cholinergic agonist carbachol using whole-cell patch recording in vitro. Cellular properties studied included spike frequency adaptation, SMPOs, input resistance, sag ratio, and persistent firing. We find that spike frequency adaptation is stronger in ventral compared to dorsal MEC, and the amplitude and the duration of the medium after hyperpolarization (mAHP; [24], [25]) both vary systematically along the DV axis along with input resistance and SMPO frequency. None of these properties showed a clear difference along the ML axis. More than half (54%) of the layer II neurons showed long-lasting (>30 s) persistent firing in carbachol. The frequency of persistent firing was higher in the lateral part of MEC and did not change systematically along the DV axis. We discuss the possible roles of persistent firing of MEC layer II neurons in providing the excitatory drive necessary for grid cell firing. In addition, we discuss that dorso-ventral differences in spike frequency adaptation are opposite from the previous prediction by a computational model, suggesting that alternative mechanisms might contribute to differences in grid cell spacing along the DV-axis.

## Materials and Methods

### Slice Preparation

All recordings reported in this paper were obtained at Boston University, Boston, MA. Some of the recordings reported here were previously used in Yoshida et al. [26] for the analysis of the effect of membrane potential on the SMPO frequency. All experimental protocols were approved by the Institutional Animal Care and Use Committee at Boston University (permit number: 07–015). Long-Evans rats of either sex (postnatal days 17 to 25; Charles River, Wilmington, MA) were deeply anesthetized with isoflurane (Abbot Laboratories). After the absence of both pedal and tail pinch reflex was confirmed, the brain was removed and placed in ice-cold artificial cerebrospinal fluid (ACSF) containing (in mM) 124 NaCl, 3 KCl, 1.25 NaH_2_PO_4_, 26 NaHCO_3_, 1.6 CaCl_2_, 1.8 MgSO_4_, 10 glucose (pH adjusted to 7.4 by saturation with 95% O_2_ - 5% CO_2_). 350 µm-thick slices were cut horizontally using a tissue slicer (World Precision Instruments Vibroslicer or Leica VT 1000). After cutting, the slices were transferred to a holding chamber, where they were kept submerged at 30 degrees for 30 min and then at room temperature for at least another 30 more min before transfer to the recording chamber. The holding chamber was filled with the ACSF.

### Electrophysiological Recording

Slices were transferred to a submerged recording chamber and superfused with ACSF, maintaining the temperature between 34 to 36°C for recordings. Patch pipettes were fabricated from borosilicate glass capillaries by means of a P-87 horizontal puller (Sutter Instrument). Patch pipettes were filled with intracellular solution containing (in mM) 120 K-gluconate, 10 HEPES, 0.2 EGTA, 20 KCl, 2 MgCl, 7 phosphocreatine-diTris, 4 Na_2_ATP and 0.3 TrisGTP (pH adjusted to 7.3 with KOH). The intracellular solution also contained 0.1% biocytin for the purpose of labeling. When filled with this solution, the patch pipettes had a resistance of 3–5 MΩ. Slices were visualized with an upright microscope (Zeiss Axioskop 2), equipped with a ×40 water-immersion objective lens, and a near-infrared charge-coupled device (CCD) camera (JAI CV-M50IR). Tight seals (>1 GΩ) were formed on cell bodies and the membrane was ruptured with negative pressure. Current-clamp recordings were made with a Multi Clamp 700B amplifier (Axon Instruments) using built-in bridge balance and capacitance compensation circuits. Signals were lowpass filtered at 5 kHz or 10 kHz and sampled at 10 kHz or 20 kHz, respectively, using Clampex 9.0 software (Axon Instruments). A liquid junction potential of 10 mV was not corrected.

Stock solutions of carbachol (10 mM, in water) was prepared and diluted 1000 times in the ACSF. Kynurenic acid and picrotoxin were directly dissolved in the ACSF. Chemicals were purchased from Sigma-Aldrich and Tocris Bioscience. All the recordings were done in the presence of kynurenic acid (2 mM) and picrotoxin (100 µM) to suppress ionotropic glutamate receptors and GABAA receptors, respectively.

AHP currents can rundown during whole-cell patch clamp recordings [27]. Recordings of AHP in our experiment were conducted 11±4 min after break-in to the cell (range: 5–21 min; n = 33) in normal ACSF, and 24±9 min after break-in (range: 8–47 min; n = 29) in carbachol. The AHP amplitude as a function of time from break-in did not show significant decrease in AHP amplitude (data not shown). In addition, break-in time in dorsal and ventral cells was not different (data not shown).

### Data Analysis

Matlab® (The MathWorks Inc., Version 7.9, 2009) was used for data analysis.

The single exponential fit for the spike frequency adaptation was performed using the exp2fit function in Matlab. The instantaneous firing frequency was fitted with the equation *A+Be^ (−t/τ)^* where *t* is time, *A* and *B* are fitting parameters. We report the value *B* as the amplitude of exponential component and the value τ as the time constant.

The frequency of SMPOs in each cell was computed as follows. The membrane potential was depolarized with small stepwise increases in current injection (of magnitude 20 to 100 pA) from near resting potential to firing threshold progressively over a period of about three minutes. Once the firing threshold was reached and the cell was firing continuously at low frequency (<1 Hz), the experiment was terminated. Using the Fast Fourier Transform (FFT), the membrane potential recordings were analyzed with a 6.56 second-long window that shifted continuously across this membrane potential trace. Then, we chose 3 windows with the largest FFT power between 2 and 30 Hz and obtained the average peak frequency and power of these 3 windows. These three windows were always detected from the most depolarized membrane potential where strong SMPOs were observed.

Sag potential was analyzed using the voltage deflection of membrane potential by a 1 s, 400 pA hyperpolarization current from the baseline potential of −60 mV. The steady state potential for sag was measured as an average membrane potential during the last 200 ms period of current injection. Sag ratio was calculated by dividing the steady state potential by the peak potential.

Significance level <0.05 (ns: not significant, *: P<0.05, **: P<0.01, ***: P<0.001) was used. Data are expressed as means ± SEM.

### Identification of Anatomical Location

**Figure 1:**
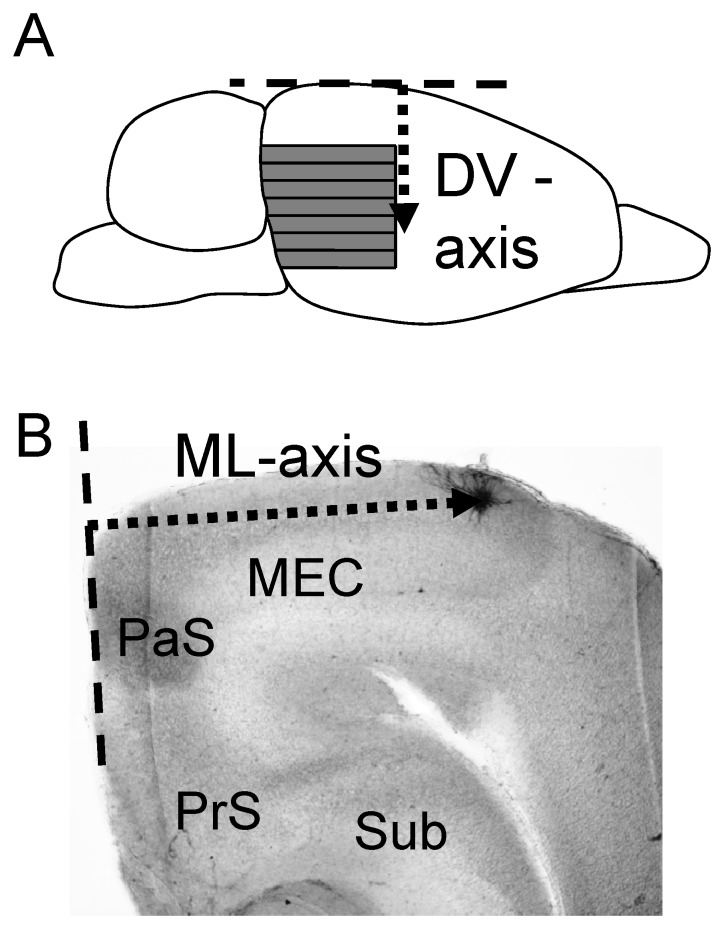
Measurements of anatomical locations of medial entorhinal cortex layer II neurons. (A) Measurement along the dorso-ventral (DV) axis. In the schematic drawing of a rat brain, each gray strip corresponds to one brain slice. The arrow indicates the distance from the cortical surface to the slice, which was used as the location of the recorded neuron along the DV-axis. (B) Measurement along the medio-lateral (ML) axis. On the photo of the brain slice, the edge of the slice along the parasubiculum (PaS) was first determined (dotted lines). The arrow indicates the distance from the dotted lines to the recorded neuron, which was used as the location of the recorded neuron along the ML-axis. PrS: Presubiculum. Sub: Subiculum.

Distances of recorded neurons from the dorsal surface were determined by keeping track of the position of each slice with separate holding chambers. At the end of slicing, the distance from the dorsal surface to the last slice was measured with a ruler. The anatomical location of the cell on the DV axis was obtained from this measure. Figure 1A describes the calculated DV measure by an arrow.

Locations of the cells in brain slices were confirmed by biocytin staining after recording in 87% of the cells (Fig 1B). Locations of the rest of the cells were confirmed by photos taken after recordings with the pipette still attached to the cell through the low magnification objective lens which was enough to approximate anatomical location of the cell. Figure 1B shows the way we measured the anatomical location of the cell on the ML axis. First, we determined the edge of the slice near the parasubiculum (PaS) as shown by dotted lines. The distance of the neuron from this edge was used as the anatomical location of the cell along the ML axis. Therefore, the ML axis we used in this paper is not the absolute value from the midline of the brain.

## Results

### Spike Frequency Adaptation

**Figure 2:**
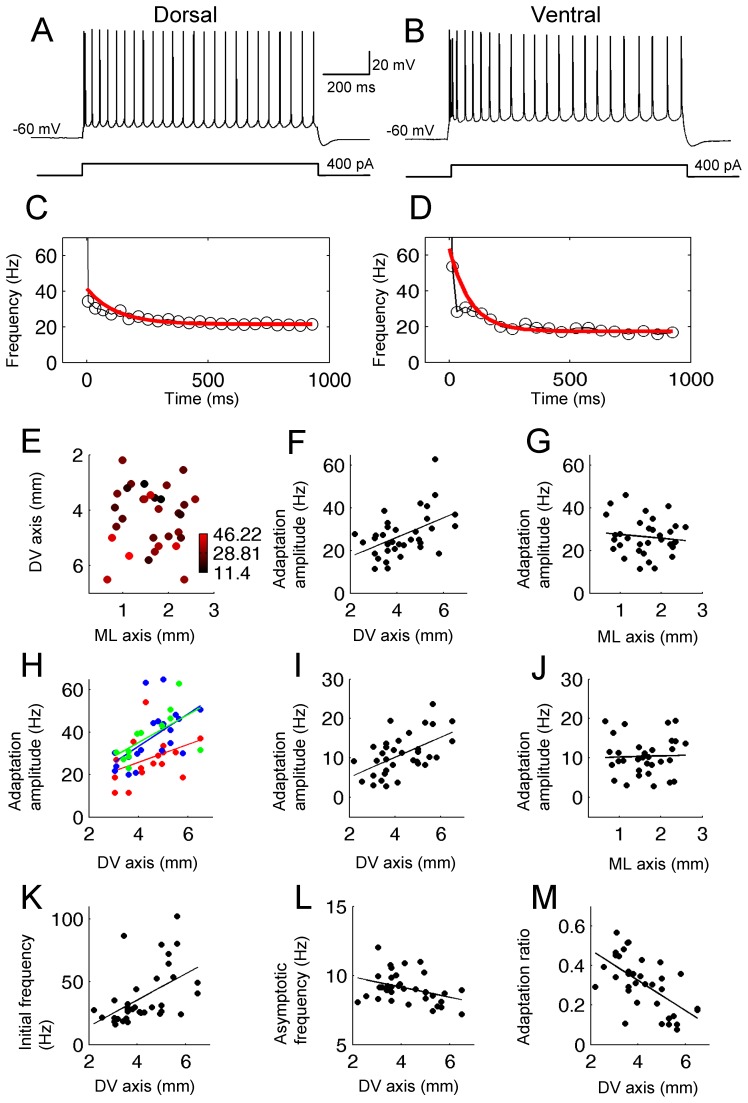
Spike frequency adaptation is greater in ventral cells in normal ACSF. (A and B) Train of action potentials elicited in a dorsal (A) and ventral (B) neuron during 1 s pulse injection. (C and D) Instantaneous frequency of neuron in A and B, respectively (black). The red line shows the single exponential fit. (E) Color plot of the adaptation amplitude (see Methods) at different locations in the two-dimensions (DV and ML) of anatomical space. Note many red dots in ventral and many black dots in dorsal locations indicating greater adaptation in ventral cells. (F and G) Adaptation amplitude plotted along the DV and ML axis, respectively. (H) Adaptation amplitude with 300 (red), 400 (green), and 500 pA (blue) current injection. (I and J) Adaptation amplitude without the first two spikes along the DV and ML axis, respectively. (K) Initial firing frequency plotted against the DV axis. (L) Asymptotic firing frequency plotted against the DV axis. (M) Adaptation ratio calculated from the initial and asymptotic frequency. Note smaller adaptation ratio in ventral cells which indicates stronger adaptation.

We first tested the anatomical distribution of firing differences in the adaptation properties of layer II MEC cells along the DV and ML axis. Spike frequency adaptation was tested by injecting a 1 s square current pulse with various amplitudes from a baseline potential of −60 mV (Fig 2 A and B). This current injection was tested multiple times by increasing the amplitude by steps of either 30 or 100 pA. To address the difference in adaptation in cells from different locations, we performed different analyses.

In the first analysis, we used the minimum current amplitude which elicited at least 20 spikes during the 1 s pulse in each cell. In this method, numbers of induced spikes were similar, while the current amplitudes were different from cell to cell. This method was used because number of elicited spikes which could systematically vary due to the gradient of input resistance along the DV axis may impact the degree of adaptation. The current amplitude was not significantly different along the DV (Fig S1 A and C) or the ML axes (Fig S1 B and D; see figure caption for statistical analysis). However, the current amplitude showed a non-significant trend along the DV axis: Current injection was larger in dorsal cells than in ventral cells in agreement with different input resistance along the DV axis.

Figures 2A and B show representative voltage responses in cells from dorsal and ventral locations, respectively. Instantaneous spiking frequencies from these traces are plotted by black lines in Fig 2C and 2D, respectively. Spike frequency adaptation was more significant in ventral cells (Fig 2B) than in dorsal cells (Fig 2A). To quantitatively address this difference, instantaneous spike frequency was fitted with a single exponential function (red lines in Fig 2C and D; See methods for detail of the fitting procedure.).

Fig 2E shows the amplitude of the exponential component of all cells at different two dimensional (ML-DV) anatomical positions across the extent of medial entorhinal cortex (n = 33). Each dot corresponds to the anatomical location of the cell and the color codes adaptation amplitude. Adaptation amplitude was clearly larger (red) in the cells located in the ventral part than in the dorsal part of the MEC layer II (Fig 2E). This can also be seen in Fig 2F where adaptation amplitudes are plotted along the DV axis. A significant linear correlation of adaptation amplitude was found along the DV axis (linear fit, R = 0.48, P<0.01, n = 35). On the other hand, the adaptation amplitude did not systematically change along the ML axis (Fig 2E and G, linear fit, R = 0.12, P = 0.53, n = 35). In this analysis, the average number of spikes during the current injection in the dorsal cells (DV <4 mm) and ventral cells (DV >4 mm) were similar (22.2±0.51 in dorsal and 22.2±0.66 in ventral cells; T-test, p = 0.93).

In the second analysis, we compared spike frequency adaptation of dorsal and ventral cells using the same current injection amplitudes among cells. We used this method to confirm findings above with a more conventional method. Figure 2H shows the adaptation amplitudes at three different current intensities, 300 (red), 400 (green), and 500 pA (blue), respectively. It is seen that the systematic change in adaptation along the DV axis exists for all three current intensities. The linear fit was significant when the current injection was 400 and 500 pA (R = 0.39, P = 0.17, n = 17 with 300 pA; R = 0.58, P<0.01, n = 21 with 400 pA; R = 0.62, P<0.05, n = 12 with 500 pA). We did not observe a systematic difference along the ML axis at any of the current intensities (data not shown).

As the third analysis, we performed the single exponential fit of the instantaneous spiking frequency without the first two spikes. This analysis was done because some of the cells showed high-frequency (>50 Hz) duplet or triplet spiking only at the beginning of the current injection, and such high frequency firing might have influenced the systematic difference along the DV axis. As shown in the Fig 2I, the adaptation amplitude increased along the DV axis indicating a stronger adaptation in ventral cells even when the first two spikes were removed (R = 0.54, P<0.01, n = 34). However, there was no systematic change along the ML axis (Fig 2J; R = 0.03, P = 0.86, n = 34).

In addition, we analyzed the spike frequency adaptation using the difference of the initial and the asymptotic spiking frequencies during the 1 s current injection. The initial and the asymptotic firing frequencies were determined as the average of the first two instantaneous frequencies and the average of the last two instantaneous frequencies, respectively. As shown in Fig 2K, the initial firing frequency was higher in the ventral cells than in the dorsal cells (R = 0.52, P<0.01, n = 35). On the other hand, the asymptotic frequency was higher in the dorsal cells than in the ventral cells as shown in the Fig 2L (R = 0.37, P<0.05, n = 35). Based on these two measures, we calculated the adaptation ratio as the asymptotic frequency divided by the initial frequency. The adaptation ratio was larger in dorsal and smaller in ventral cells, indicating stronger adaptation in the ventral cells as shown in the exponential fit analysis (Fig 2M; R = 0.62, P<0.001, n = 35).

The medial entorhinal cortex layer II contains at least two major types of neurons: stellate and non-stellate cells [28]. To examine whether the anatomical distribution of different types of neurons contributes to our observation, we divided our cells into stellate-like and non-stellate-like cells. Since the stellate cells have larger voltage sag potentials and larger SMPO amplitudes [28], we used these two measures to categorize our cells. First, we used sag ratio >0.15 and SMPO power >0.0015 mV^2^/Hz as in our previous paper [26]. This gave 26 stellate-like cells and 5 non-stellate-like cells. Stellate-like cells alone showed decreased adaptation ratio (data not shown; Linear fit R = −0.66, P<0.001, n = 26) towards the ventral direction as in the case of all cells. Next, we further tested more conservative criteria to categorize cells as stellate cells. This was to eliminate the possibility that the set of criteria used above classified some non-stellate cells as stellate cells, and the dorso-ventral difference observed within the stellate cell group was due to non-stellate cells included in the stellate group. We therefore used sag ratio >0.3 and SMPO power >0.003 mV^2^/Hz, which divided our population into 16 stellate-like cells and 15 non-stellate-like cells. Interestingly, both of these cell types were distributed equally to dorsal and ventral halves of the MEC (Stellate-like group, 8 in dorsal and 8 in ventral half; non-stellate-like group, 8 in dorsal and 7 in ventral half). In addition, not only stellate-like cells alone but also non-stellate cells alone showed decreased adaptation ratio (data not shown; stellate cells, linear fit R = −0.61, P<0.05, n = 16; non-stellate cells, linear fit R = −0.64, P<0.05, n = 15) towards ventral direction. These results suggest that stronger spike frequency adaptation in ventral cells in our dataset is not due to different anatomical distribution of stellate and non-stellate cells along the DV axis.

**Figure 3:**
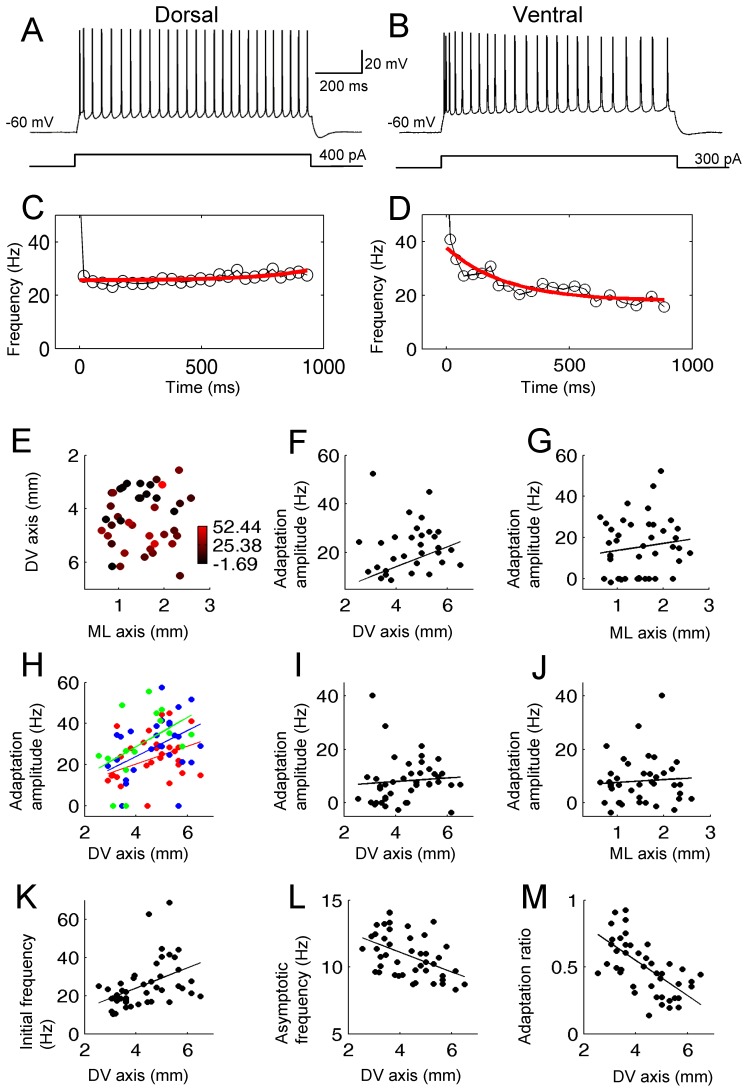
Spike frequency adaptation is greater in ventral cells in carbachol. (A and B) Train of action potentials elicited in a dorsal (A) and a ventral (B) neuron during 1 s pulse injection. (C and D) Instantaneous frequency of neuron in A and B, respectively (black). The red line shows the single exponential fit. (E) Color plot of the adaptation amplitude at different locations in the two-dimensions (DV and ML) of anatomical space. Note many red dots in ventral and many darker dots in dorsal locations indicating greater adaptation in ventral cells. (F and G) Adaptation amplitude plotted along the DV and ML axes, respectively. (H) Adaptation amplitude with 300 (red), 400 (green) and 500 pA (blue) current injection. (I and J) Adaptation amplitude without the first two spikes along the DV and ML axis, respectively. (K) Initial firing frequency plotted against the DV axis. (L) Asymptotic firing frequency plotted against the DV axis. (M) Adaptation ratio calculated from the initial and asymptotic frequency.

To assess the effect of cholinergic activation on this spatial distribution of the spike frequency adaptation within the MEC layer II, we analyzed the degree of adaptation in the presence of 10 µM carbachol in the same set of cells. In the presence of carbachol (10 µM), the adaptation was reduced in agreement with previous studies in entorhinal cortex [29] as well as other cortical structures [30], [31]. However, the anatomical gradient of the adaptation index along the DV axis was similar to that in the normal ACSF. With the first analysis with the single exponential fits, the color plot showed a gradient along the dorso-ventral direction (Fig 3E; n = 42) and the adaptation amplitude plotted on the DV-axis showed greater adaptation in ventral cells (Fig 3F; R = 0.31, P<0.05, n = 44). The adaptation amplitude did not systematically change along the ML-axis (Fig 3G; R = 0.14, P = 0.39, n = 44). This gradient was also clear with the second analysis using absolute current (Fig 3H; R = 0.35, P = 0.05, n = 31 with 300 pA; R = 0.41, P<0.05, n = 30 with 400 pA; R = 0.47, P = 0.08, n = 15 with 500 pA). The gradient was however not significantly correlated along the DV axis when the first 2 spikes were eliminated (Fig 3I; R = 0.08, P = 0.61, n = 42).

In the analysis with the initial and the asymptotic frequency, the initial firing frequency was higher in the ventral cells (Fig 3K, R = 0.44, P = 0.003, n = 44) and the asymptotic frequency was higher in the dorsal cells (Fig 3L; R = 0.47, P<0.01, n = 44). The adaptation ratio calculated from these two frequencies was larger in dorsal and smaller in ventral cells, indicating stronger adaptation in the ventral cells as in the case of normal ACSF (Fig 3M; R = 0.63, P<0.001, n = 44).

In summary, adaptation showed a gradient along the DV axis both with and without cholinergic activation.

### After Hyperpolarization Potential

The medium after hyperpolarization potential (mAHP) is known to contribute to spike frequency adaptation [25], [32]–[34]. Therefore, a systematic change in the mAHP along the DV axis might be underlying the differences in spike frequency adaptation observed in the dorsal and ventral locations. In fact, it has been reported that the mAHP duration is longer in ventral cells than in dorsal cells in adult rats (P34–P56) and the mAHP amplitude is larger in dorsal cells than in ventral cells in young rats (P17–P25; [13]). It has also been reported that the time constant of the mAHP is longer in ventral cells [19], [35]. However, these previous studies did not report whether these mAHP parameters are correlated with the spike frequency adaptation. In addition, the effect of cholinergic modulation was not studied in these previous studies.

**Figure 4:**
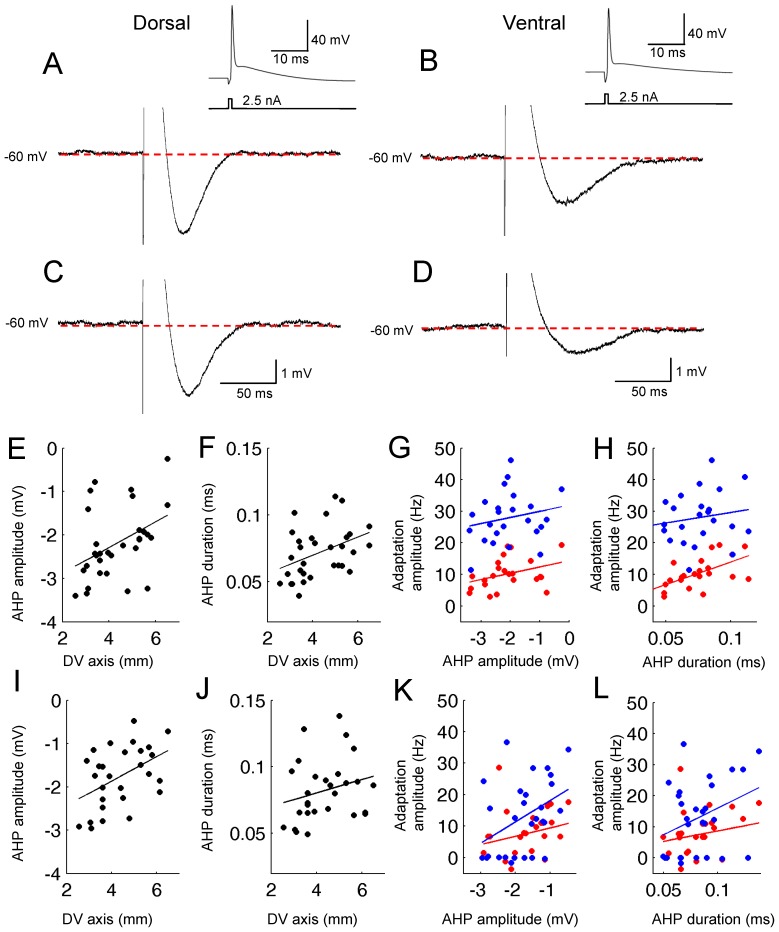
Spike AHP duration and amplitude systematically change along the DV axis. (A and B) Examples of AHP in the normal ACSF in a dorsal (A) and a ventral cell (B). The AHP was induced by one action potential elicited by a 1 ms current injection from the baseline membrane potential of −60 mV. Inset shows this action potential. (C and D) Examples of AHP in carbachol (10 µM) in a dorsal (C) and a ventral cell (D). (E) Amplitudes of AHP in normal ACSF plotted on the DV axis. (F) Duration of AHP in normal ACSF plotted on the DV axis. (G) Correlation between the amplitude of AHP and the adaptation amplitude in normal ACSF. Adaptation amplitudes of exponential fit with and without the first two spikes are plotted with blue and red dots, respectively. The color code is the same for all other plots. (H) Correlation between the duration of AHP and the adaptation amplitude in normal ACSF. (I) Amplitudes of AHP in carbachol plotted on the DV axis. (J) Duration of AHP in carbachol plotted on the DV axis. (K) Correlation between the amplitude of AHP and the adaptation amplitude in carbachol. (L) Correlation between the duration of AHP and the adaptation amplitude in carbachol.

In the current study, we studied the amplitude, duration and the area of an mAHP elicited by a single action potential (inset of Fig. 4A and B) using a 1 ms current injection at −60 mV. The measurements of the mAHP parameters were taken relative to the unique baseline (−60 mV). We employed this method to eliminate the possible effects of different baseline membrane potential and/or different spiking frequencies from cell to cell on the mAHP parameters in earlier studies [13], [35].

Figure 4A and B, show an AHP induced by single action potential at −60 mV in a dorsal and a ventral neuron, respectively. The amplitude of the mAHP is larger in the dorsal than in the ventral cell. On the other hand, the duration of the mAHP (see Methods) was longer in the ventral than in the dorsal cell. Figure 4E and F show that this was significantly correlated to the DV axis of the MEC where the amplitude decreased along the DV axis (Fig 4E; R = 0.42, P<0.05, n = 31) while the duration increased along the DV axis (Fig 4F; R = 0.40, P<0.05, n = 31). We then asked if the amplitude and/or the duration correlate with the spike frequency adaptation observed above. In Fig 4G, adaptation amplitude from the single exponential fit either with (blue) and without (red) the first two spikes is plotted against the mAHP amplitude of the cell. We found a non-significant trend where the adaptation amplitude was larger in cells with smaller mAHP amplitude either with or without the first two spikes (Fig 4G; R = 0.2, P = 0.34, n = 30 with the first two spikes; R = 0.35, P = 0.1, n = 29 without the first two spikes). On the other hand, the adaptation amplitude was significantly correlated with the duration of the mAHP when the first two spikes were omitted (Fig 4H red, R = 0.6, P<0.05, N = 29) but was not significantly correlated when the first two spikes were included (Fig 4H blue, R = 0.17, P = 0.44, n = 30). We did not find any clear trend along the ML axis in these mAHP parameters (data not shown).

In the presence of carbachol, the amplitude and the duration of the mAHP along the DV axis showed a similar difference as in the control condition although the trend of the duration of mAHP along the DV axis did not reach significance (Fig. 4I and J; R = 0.46, P<0.05, n = 28; R = 0.24, P = 0.2, n = 28). The correlation of these values with the adaptation amplitude showed a similar trend as in the normal ACSF condition in both of the parameters. First, cells with a larger mAHP amplitude showed a smaller adaptation amplitude when the first two spikes were included (Fig. 4K blue; R = 0.40, P<0.05, n = 39) while there was no significant correlation when first two spikes were not included (R = 0.23, P = 0.26, n = 37). Second, cells with a longer mAHP duration had a tendency to show a larger adaptation slope although the linear correlation was not significant either with (Fig 4L blue; R = 0.20, P = 0.32, n = 37) or without (Fig 4L red; R = 0.34, P = 0.08, n = 39) the first two spikes. We did not find a clear trend along the ML axis in any of these measurements in carbachol (data not shown).

In summary, we found that the mAHP amplitudes of MEC layer II neuron decreases while the duration of the mAHP increases along the DV axis, in agreement with the previous studies [13], [35]. In addition, we found that the longer duration of mAHP may contribute to more spike frequency adaptation.

### Subthreshold Membrane Potential Oscillations (SMPOs)

**Figure 5:**
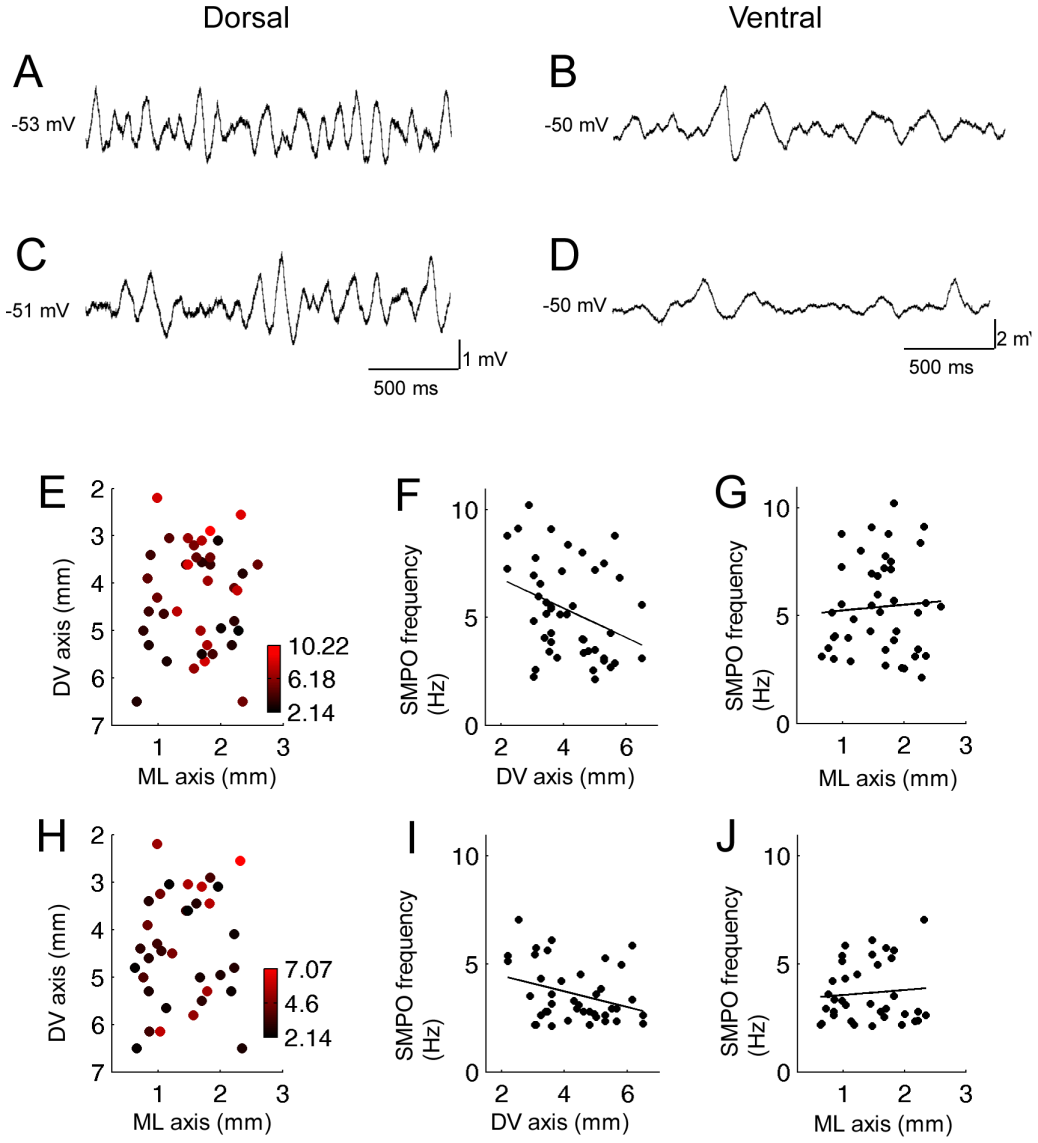
Frequency of subthreshold membrane potential oscillations change systematically along the DV but not along the ML axis. (A and B) Examples of SMPOs in the normal ACSF in a dorsal (A) and a ventral cell (B). The SMPO frequency was higher in dorsal cells than in ventral cells. (C and D) Examples of SMPOs in carbachol (10 µM) in a dorsal (C) and a ventral cell (D). (E) Color plot of the SMPO frequency at different positions in two-dimensions (DV and ML) of anatomical space in normal ACSF. (F and G) SMPO frequency along the DV and ML axis, respectively, in normal ACSF. (E) Color plot of the SMPO frequency at different positions in the two-dimensions (DV and ML) of anatomical space in carbachol. (F and G) SMPO frequency along the DV and ML axis, respectively, in carbachol.

To study the distribution of the SMPO frequency along the DV and ML axes, the membrane potential was depolarized by increasing the DC current injection to the level just below the spike threshold where the largest SMPOs amplitude was observed. Figure 5A and B show examples of SMPOs in a dorsal and a ventral neuron, respectively. It is seen that the dorsal cell shows a SMPOs with relatively high frequency (∼9 Hz) while the ventral cell shows SMPOs with a lower frequency (∼3 Hz). Figure 5E shows a two-dimensional anatomical space of the MEC layer II, where the frequency of SMPOs from each cell is shown by the color of an individual dot (n = 42). Higher frequency is shown by red while lower frequency is shown by black dots. While red dots which can be seen in the dorsal parts gradually disappear toward the more ventral part such a gradient is not seen on the ML axis. When the frequency is plotted along the DV axis, SMPO frequency linearly decreases along the DV axis as reported earlier (Fig 5F; R = 0.34, P<0.05, n = 45; [6],[36]). On the other hand, SMPO frequency did not show a systematic change along the ML axis (Fig 5G; R = 0.064, P = 0.69, n = 45).

To assess the effect of cholinergic activation on this spatial distribution of SMPO frequency within the MEC layer II, we measured the SMPO frequency in the presence of 10 µM carbachol in the same set of cells. As reported earlier [23], carbachol decreased the frequency of SMPO (Fig 5C and D). The anatomical distribution of the frequency on the 2D space showed a similar but less clear trend compared to that in the normal ACSF condition (Fig 5H; n = 37). Analysis along the DV axis also showed a similar significant but lesser degree of gradient compared to the normal ACSF condition mentioned above (Fig 5I; R = 0.32, P = 0.039, n = 41). The SMPO frequency was similar along the ML axis and did not show systematic differences (Fig. 5J; R = 0.087, P = 0.61, n = 41).

### Sag Ratio

**Figure 6:**
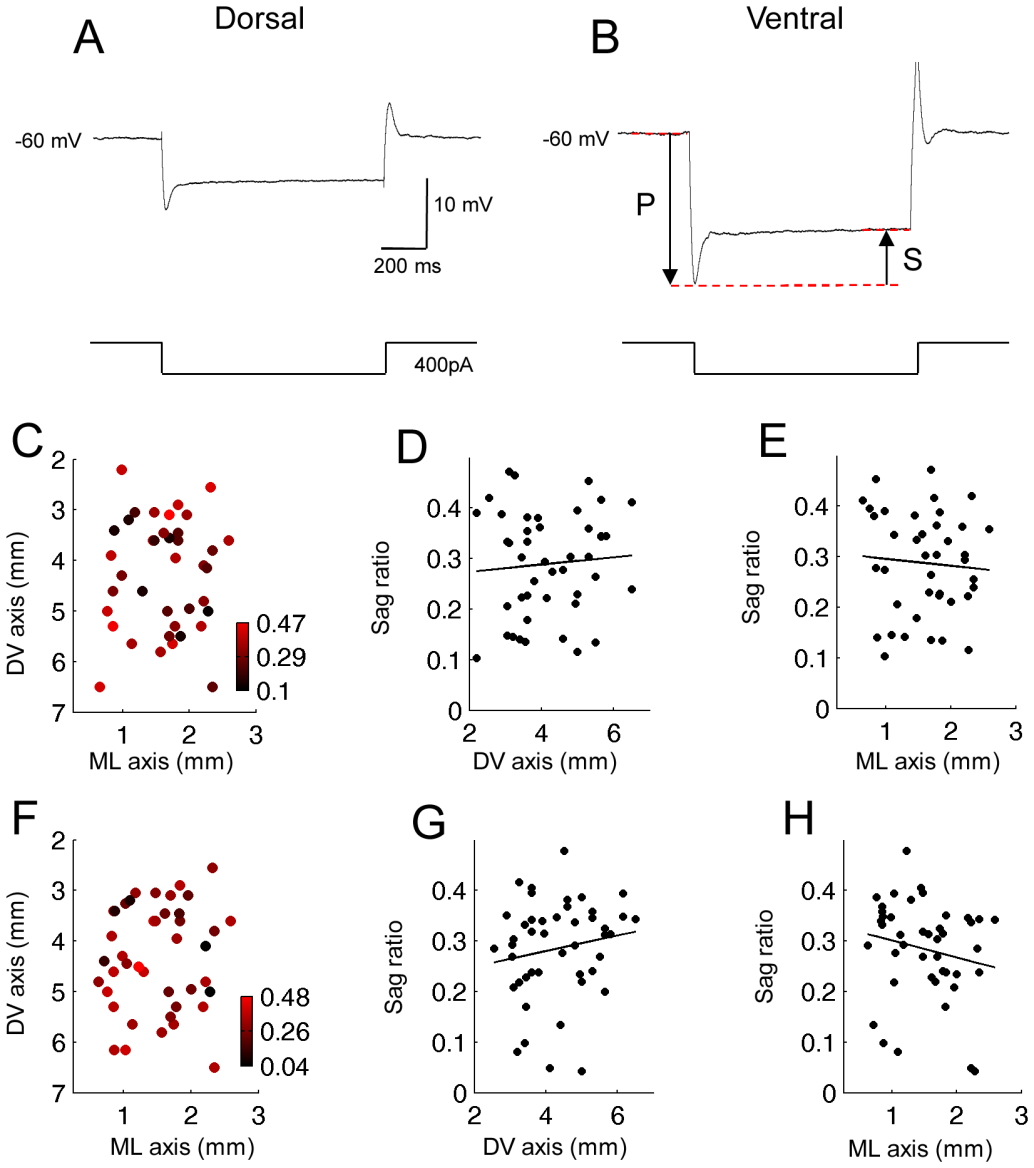
Sag potential does not systematically change along the DV and ML axis. (A and B) Examples of sag potential in the normal ACSF in a dorsal (A) and a ventral cell (B). Sag ratio was calculated by dividing the steady state potential (indicated by arrow S) by the peak potential (indicated by arrow P). The sag ratios were similar in dorsal and in ventral cells. (C) Color plot of the sag ratio at different positions in the two-dimensions (DV and ML) of anatomical space in normal ACSF. (D and E) Sag ratio along the DV and ML axis, respectively, in normal ACSF. (F) Color plot of the sag ratio at different positions in the two-dimensions (DV and ML) of anatomical space in carbachol (10 µM). (G and H) Sag ratio along the DV and ML axis, respectively, in carbachol.

We tested if the sag potential is distributed differently along the DV and ML axes, both in normal ACSF and with the presence of carbachol. Figure 6A and B show examples of the sag potential measured in a dorsal and a ventral MEC layer II neuron. Sag potential was induced by 400 pA hyperpolarization current injection from the baseline membrane potential of −60 mV. Sag ratio was calculated by dividing the size of the sag potential (indicated by S) by the peak voltage deflection (indicated by P; Fig. 6B; see Methods for detail).

Both in normal ACSF (Fig 6C, n = 41; Fig 6D, R = 0.08, P = 0.6, n = 45; Fig 6E, R = 0.07, P = 0.66, n = 45) and in carbachol (Fig 6F, n = 43; Fig 6G, R = 0.16, P = 0.29, n = 45; Fig 6H, R = 0.19, P = 0.23, n = 45), the sag ratio showed no significant gradient along the DV or ML axis.

### Input Resistance

The input resistance is a good measure of the excitability of neurons. It has been reported that the input resistance is larger in ventral cells than in dorsal cells in MEC layer II in the mouse and the rat [9], [13]. Here, we first investigated the input resistance gradient in two anatomical dimensions. Second, we tested the effect of cholinergic activation on the input resistance. In all conditions, input resistance was measured using the membrane potential deflection induced by a brief negative current injection (500 ms, 100 pA) from a membrane potential of −60 mV.

**Figure 7:**
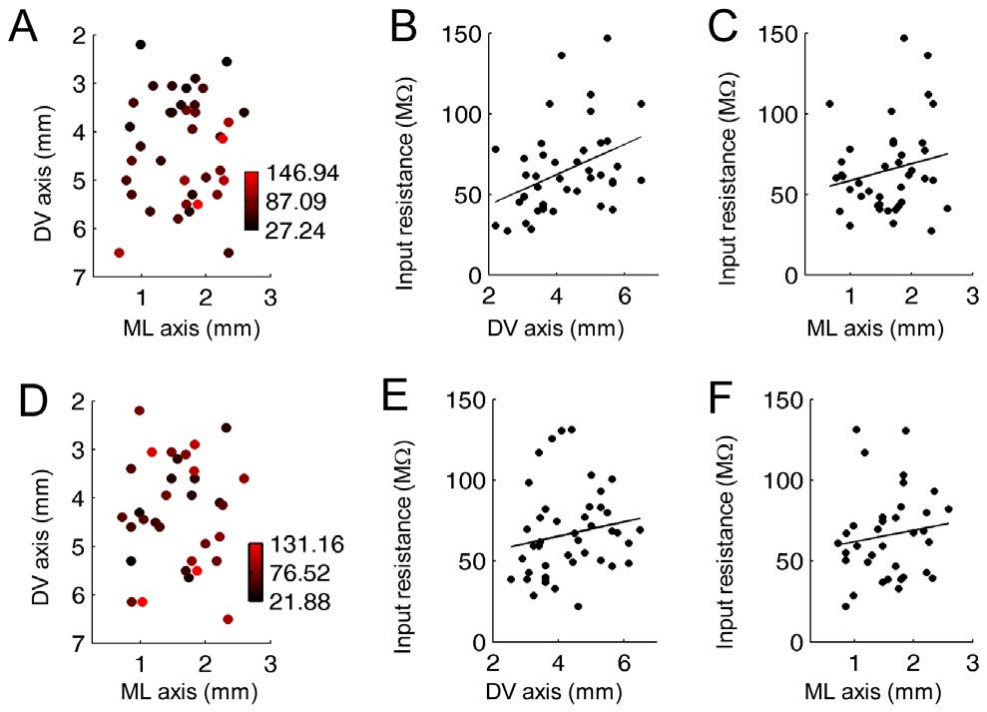
Input resistance systematically changes along the DV axis. (A) Color plot of the input resistance at different positions in the two-dimensions (DV and ML) of anatomical space in normal ACSF. (B and C) Input resistance along the DV and ML axis, respectively, in normal ACSF. (D) Color plot of the input resistance at different positions in the two-dimensions (DV and ML) of anatomical space in carbachol (10 µM). (E and F) Input resistance along the DV and ML axis, respectively, in carbachol.

Our analysis showed that the input resistance in normal ACSF condition was larger in ventro-lateral parts than other locations within the two anatomical dimensions (Fig 7A; n = 40). The input resistance was larger in ventral neurons than in dorsal neurons when plotted on the DV axis as previously reported (Fig 7B; R = 0.39, P<0.05, n = 45). In addition, an increasing trend was also observed along the ML axis although the linear fit was not significant (Fig. 7C; R = 0.20, P = 0.22, N = 44).

In the presence of 10 µM carbachol, the above mentioned gradient across two anatomical dimensions was less clear (Fig 7F; n = 42). The increasing trend along the DV (Fig 7G; R = 0.17, P = 0.28, n = 45) and ML axis were both less clear than in the normal ACSF condition (Fig 7H; R = 0.13, P = 0.45, N = 44).

### Persistent Firing

**Figure 8:**
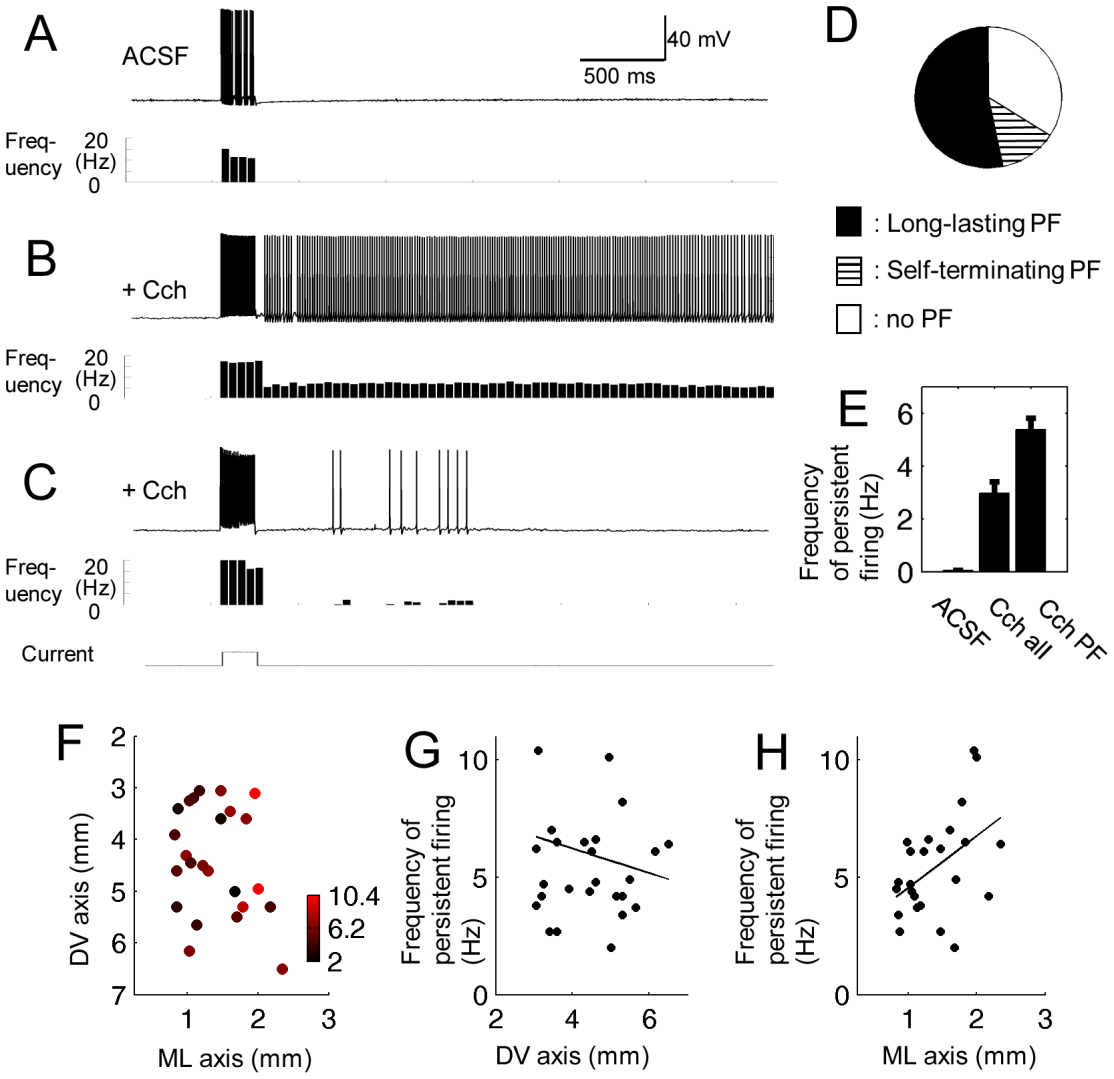
Persistent firing can be induced in cholinergic receptor agonist. (A) Response of an example cell to a 2 s current injection (100 pA) in normal ACSF. Histogram below shows firing frequency. (B) Response of the same cell to the same stimulation in carbachol (10 µM). Long-lasting persistent firing lasting for more than 30 s was elicited by the stimulation. (C) Example of self-terminating persistent firing (<30 s) in carbachol in a different cell. (D) Ratio of cells that showed long-lasting persistent firing, self-terminating persistent firing, and no persistent firing in carbachol. (E) Average frequency of persistent firing in normal ACSF and in carbachol. The bar graphs indicated by “Cch all” and “Cch PF” show average frequency of persistent firing in all cells and only in cells that showed long-lasting persistent firing, respectively. (F) Color plot of the frequency of persistent firing of cells that showed long-lasting persistent firing on the two-dimensional (DV and ML) anatomical space in carbachol. (G and H) Frequency of persistent firing of cells that showed persistent firing along the DV and ML axis, respectively, in carbachol. Cch: carbachol. PF: persistent firing.

Persistent firing in MEC layer II cells has not been described intensively and the anatomical distribution of the persistent firing property is unknown. To test persistent firing properties in MEC layer II cells, membrane potential was depolarized to just below firing threshold by a DC current injection. Then, brief depolarization current (2 s, 100 pA) was injected into the cell from the recording pipette. This current injection elicited action potentials at around 20–30 Hz during stimulation. In normal ACSF, membrane potential went back to the baseline membrane potential and there were no further action potentials elicited (Fig 8A). Therefore, in the normal ACSF condition, no cells showed persistent firing (n = 40).

However, in the presence of 10 µM carbachol, the same stimulation caused sustained firing that lasted more than 30 seconds in 54% of cells (long-lasting persistent firing; Fig 8B; 26 out of 47). 13% of the cells showed persistent firing that terminated on its own within 30 s (self-terminating persistent firing; Fig. 8C; 6 out of 47). The rest (33%) did not show persistent firing (16 out of 47). Figure 8 D summarizes the ratio of these three different types of firing. To quantify the frequency of persistent firing, we measured the average firing frequency within a 10 s period from 10 to 20 s after termination of the stimulation in each cell. The mean frequency of the persistent firing in carbachol was 2.95±0.44 Hz (n = 47) when all cells were included and 5.37±0.42 Hz (n = 26) when only cells that showed persistent firing were included (Fig 8E).

We plotted the frequency of persistent firing using cells that showed long-lasting persistent firing (Fig 8F–H). The frequency of the persistent firing did not show systematic difference along the DV axis (Fig 8I, n = 24; Fig 8J, R = 0.14, P = 0.5, n = 26). However, the frequency was higher in the lateral part than in the medial part of the MEC (Fig 8K, R = 0.47, P<0.05, n = 26). In summary, we report long-lasting persistent firing in layer II principal cells in the MEC for the first time to the best of our knowledge. In addition, the frequency of persistent firing might differ along the ML axis.

## Discussion

In this paper, we investigated the anatomical distribution of several cellular properties of MEC layer II neurons within the two dimensional anatomical extent of MEC. We tested the anatomical distribution of these properties both with and without the cholinergic agonist carbachol. We have shown that these cellular properties differ dependent on anatomical location and are differentially modulated by carbachol.

### Comparison with Previous Studies

Our observation indicated that spike frequency adaptation shows a clear difference at different anatomical locations along the DV axis (Fig 2). To our knowledge, this is the first report of the anatomical distribution of spike frequency adaptation in the MEC. It has been shown that mAHP [25] duration is longer in ventral cells than in dorsal cells MEC layer II neurons [13], [35]. However, those studies did not report the correlation between the mAHP and spike frequency adaptation. In this paper, we found that the mAHP duration is correlated with the spike frequency adaptation, indicating that longer duration of the mAHP might contribute to stronger spike frequency adaptation in ventral cells (Fig 4).

Garden et al [9] reported that current injection necessary to induce one action potential was significantly larger in the dorsal cells. Our data showed similar but non-significant trend along the DV axis: The amplitude of current injection to induce 20 spikes in 1 s was larger in dorsal cells than in ventral cells (Fig S1 A and E). This might be due to different numbers of spikes elicited (>20 spikes) in our study and in Garden et al study (>1 spike). When only one spike is induced, there is no contribution of spike frequency adaptation. On the other hand, when multiple spikes are induced, spike frequency adaptation influences the number of spikes that can be elicited by the current injection. As we have shown in this study, spike frequency adaptation is stronger in ventral than in dorsal MEC. Therefore, while smaller input resistance requires more current injection in dorsal cells to initiate firing, smaller spike frequency adaptation supports repetitive spiking in these cells. Therefore, as the number of elicited spikes increases, the input resistance difference along the DV could be counteracted by the adaptation difference along the DV axis.

The anatomical gradient of the adaptation index along the DV axis was still present in the presence of carbachol as in normal ACSF (Fig 3) despite the fact that carbachol reduces the magnitude of spike frequency adaptation as in previous studies [29]–[31]. The retention of an anatomical gradient in carbachol is particularly interesting because other cellular properties such as SMPO frequency and input resistance, which also showed a gradient of anatomical distribution along the DV axis, showed a reduced gradient in carbachol. For example, the SMPO frequency difference between dorsal and ventral cells in carbachol was smaller than that in normal ACSF (Fig 5F and I). The input resistance gradient along the DV axis was also less clear in carbachol (Fig 7D and G).

While spike frequency adaptation, mAHP, SMPO frequency and input resistance all showed a gradient along the DV axis, we did not find a clear difference of any cellular properties along the ML axis except for the frequency of persistent firing. The lack of a significant gradient along the ML axis for SMPO frequency, sag ratio, and input resistance are in line with the previous observations [7], [37]. These authors reported that the SMPO frequency, sag ratio, and input resistance have significant correlation with the location along the ML axis only when medial entorhinal and lateral entorhinal neurons are combined but not within medial entorhinal cortex alone. This occurs due to the absence of SMPOs and sag in the lateral entorhinal cortex [7], [8], [37].

In the presence of carbachol, 54% of these layer II cells showed long-lasting (>30 s) persistent firing, 13% of cells showed self-terminating persistent firing and 33% showed no persistent firing. We believe that this is the first report of long-lasting stable persistent firing in MEC layer II neurons. Previously, Klink and Alonso (1997a) had shown that MEC layer II neurons show persistent firing in 30 µM carbachol. However, this persistent firing was observed as a repetition of long clusters of firing which lasted for 2–5 s [29]. Magistretti et al [38], has shown self-terminating persistent firing which typically stopped firing within 30 seconds using 5 to 20 µM carbachol in MEC cells. The lack of long-lasting stable persistent firing in Klink and Alonso (1997a) might be because of the higher concentration of carbachol (30 µM) used in their study than in our study (10 µM). Similarly, the lack of long-lasting persistent firing in Magistretti et al. [38] could be due to the lower recording temperature (∼22°C) employed in their study.

### Cellular Properties and Grid Cell Firing

Recent findings by Giocomo et al. [14] showed that mice with knockout of the HCN1 subunit of the h channel showed wider spacing between grid cell firing fields, but still showed a gradient of grid cell spacing along the DV axis, though these mice did not show a clear gradient of SMPOs or resonance [39]. This indicates that mechanisms other than SMPOs or resonance might also contribute to the generation of grid cell firing field spacing.

Kropff and Treves [10] proposed that firing frequency adaptation could underlie grid cell formation. That model predicted that the spacing of grid cell firing fields would increase in ventral cells if frequency adaptation is weaker in ventral cells. However, our result shows the opposite gradient where adaptation is stronger in ventral cells than in dorsal cells (Fig 2). Therefore, even though the idea is interesting, our results suggest that model does not predict the experimental observations. Different grid cell spacing along the DV axis may arise either from properties other than spike frequency adaptation, or spike frequency adaptation may affect grid cell firing in a different manner from the prediction of that model.

Here, we discuss one possible way the grid cell spacing might be modulated by differences in the degree of firing frequency adaptation along the DV axis. It has been shown that blockade of potassium currents increases the input resistance more in dorsal cells than in ventral cells [9]. This suggests that the AHP current has a stronger effect in dorsal cells and this agrees with our observation of larger AHP amplitude in dorsal cells. However, we have shown that larger AHP amplitude does not result in larger spike frequency adaptation (Fig. 4). We instead found that ventral cells that showed smaller AHP amplitudes and longer AHP durations showed stronger frequency adaptation (Fig. 2). Because of the smaller magnitude of frequency adaptation in dorsal cells, the asymptotic firing rate was higher in dorsal than in ventral cells (Fig. 2 L). This was the case even when the same amplitude of current injection was used for all cells. In fact, a linear fit showing a systematic decrease of the asymptotic firing rate was significant along the DV axis when current injection was 400 pA in normal ACSF, and at 300 and 400 pA in carbachol (data not shown). This indicates that despite their lower input resistances, dorsal cells might fire more action potentials when depolarized for a prolonged period of time. In turn, this intense firing might activate a local inhibitory network more strongly in dorsal than ventral MEC. Further, this strong inhibition might help the bump of activity to be suppressed in previous locations and move more quickly, resulting in a smaller spacing of grid cell firing fields.

Recently, in vivo intracellular recordings from grid cells have shown that grid cells undergo sustained depolarization as the animal crosses the firing field [15] which is consistent with continuous attractor models of grid cells [4], [5], [40]. On the other hand, MEC layer II is virtually devoid of excitatory recurrent connections [17] that play an important role in continuous attractor models to maintain excitatory drive to maintain firing. While the excitatory drive might alternatively be provided by nearby structures such as the hippocampus and the post-subiculum, such excitatory drive might also be provided by a cellular property such as persistent firing reported in this paper. Since the persistent firing was observed only in the presence of cholinergic receptor agonist, lack of cholinergic receptor activation may influence grid cell firing. Recent data has shown that inactivation of medial septum blocks theta oscillations and disrupts grid cell firing in the MEC [20], [21]. Since medial septum provides cholinergic projections to the MEC, the septal inactivation may have caused a lack of cholinergic receptor activation and a lack of depolarization drive for grid cell activity. This is also consistent with a recent report that lesions of the basal forebrain cholinergic system disrupt idiothetic navigation in mice [22].

We conclude that many cellular properties vary systematically along the DV axis in MEC layer II principal neurons. Cholinergic receptor activation preserves most of these distributions intact and adds extra excitability through induction of persistent firing. Cholinergic activation, therefore, may enable these properties to be combined to form grid cells in MEC layer II cells.

## Supporting Information

Figure S1 **Minimum current injection used to elicit more than 20 spikes was not significantly different along the DV and ML axis.** (A) Amplitudes of injected current in individual cells along the DV axis in normal ACSF (linear fit, R = 0.28, P = 0.1, n = 35). (B) Amplitudes of injected current in individual cells along the ML axis in normal ACSF (linear fit, R = 0.08, P = 0.66, n = 35). (C) Comparison of injected current in dorsal and ventral cells in normal ACSF (T-test, P = 0.1, t = 1.6). Dorsal and ventral cells were divided at DV = 4.5 mm. (D) Comparison of injected current in medial and lateral cells in normal ACSF (T-test, P = 0.75, t = 0.30). Medial and lateral cells were divided at ML = 1.5 mm. (E) Amplitudes of injected current in individual cells along the DV axis in carbachol (linear fit, R = 0.23, P = 0.13, n = 44). (F) Amplitudes of injected current in individual cells along the ML axis in carbachol (linear fit, R = 0.021, P = 0.90, n = 44). (G) Comparison of injected current in dorsal and ventral cells in carbachol (T-test, P = 0.17, t = 1.4). (H) Comparison of injected current in medial and lateral cells in carbachol (T-test, P = 0.25, t = 1.2). In summary, amplitude of current injection was not significantly different along the DV and ML axes, both in control and in carbachol conditions. The non-significant trend along the DV axis might reflect different input resistance along the DV axis.
(TIFF)
