# Supplementary figures and images for: Comparison of Properties of Medial Entorhinal Cortex Layer II Neurons in Two Anatomical Dimensions with and without Cholinergic Activation
Source: PLoS One. 2013 Sep 12;8(9):e73904. doi: 10.1371/journal.pone.0073904 (PMC3771974; doi:10.1371/journal.pone.0073904)

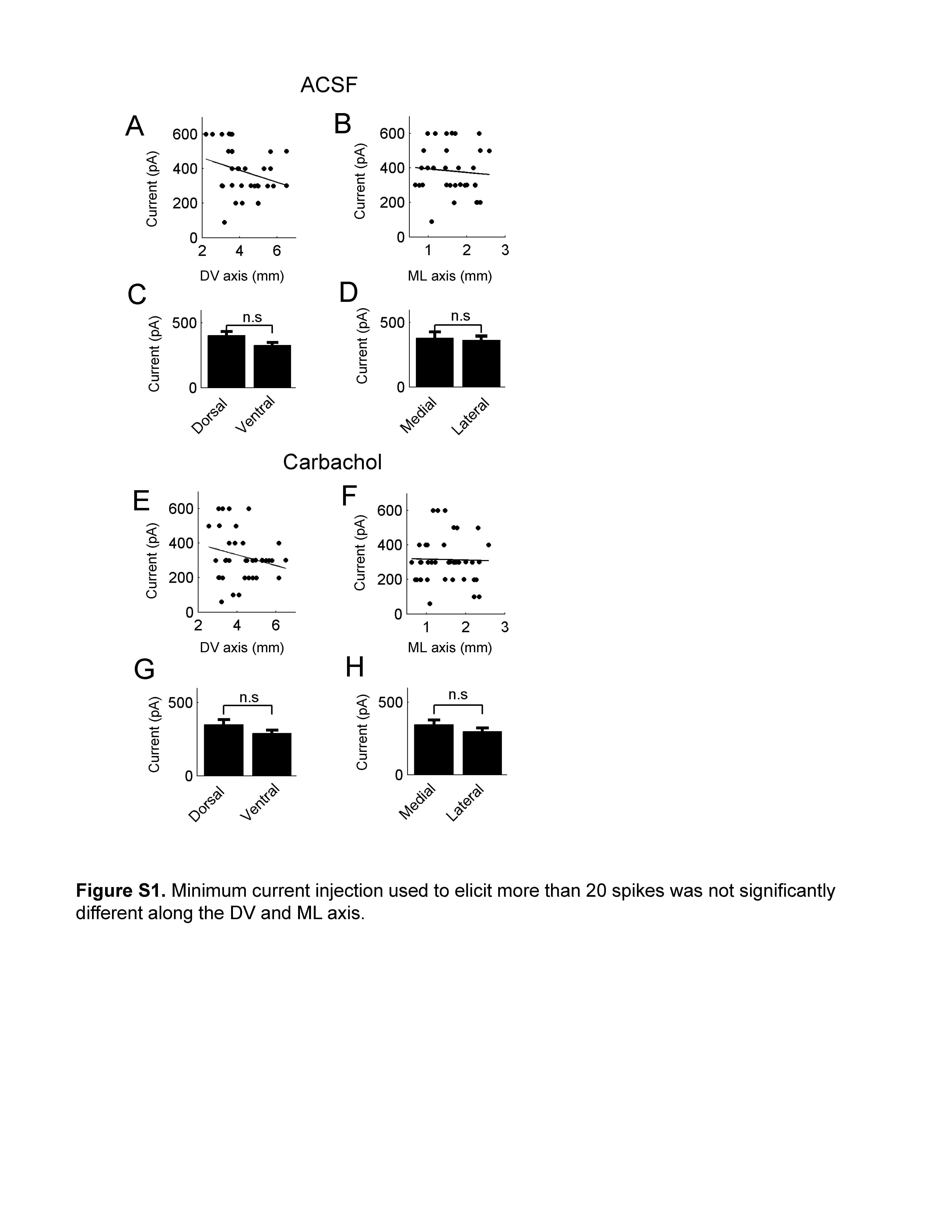

Supplement: Figure S1 — Minimum current injection used to elicit more than 20 spikes was not significantly different along the DV and ML axis. (A) Amplitudes of injected current in individual cells along the DV axis in normal ACSF (linear fit, R = 0.28, P = 0.1, n = 35). (B) Amplitudes of injected current in individual cells along the ML axis in normal ACSF (linear fit, R = 0.08, P = 0.66, n = 35). (C) Comparison of injected current in dorsal and ventral cells in normal ACSF (T-test, P = 0.1, t = 1.6). Dorsal and ventral cells were divided at DV = 4.5 mm. (D) Comparison of injected current in medial and lateral cells in normal ACSF (T-test, P = 0.75, t = 0.30). Medial and lateral cells were divided at ML = 1.5 mm. (E) Amplitudes of injected current in individual cells along the DV axis in carbachol (linear fit, R = 0.23, P = 0.13, n = 44). (F) Amplitudes of injected current in individual cells along the ML axis in carbachol (linear fit, R = 0.021, P = 0.90, n = 44). (G) Comparison of injected current in dorsal and ventral cells in carbachol (T-test, P = 0.17, t = 1.4). (H) Comparison of injected current in medial and lateral cells in carbachol (T-test, P = 0.25, t = 1.2). In summary, amplitude of current injection was not significantly different along the DV and ML axes, both in control and in carbachol conditions. The non-significant trend along the DV axis might reflect different input resistance along the DV axis. (TIFF) [file pone.0073904.s001.tiff]
